# How self-organization can guide evolution

**DOI:** 10.1098/rsos.160553

**Published:** 2016-11-16

**Authors:** Jonathan Glancy, James V. Stone, Stuart P. Wilson

**Affiliations:** 1 Department of Psychology, The University of Sheffield, Sheffield, UK; 2 Sheffield Robotics, The University of Sheffield, Sheffield, UK

**Keywords:** self-organization, natural selection, thermoregulation, huddling, endothermy

## Abstract

Self-organization and natural selection are fundamental forces that shape the natural world. Substantial progress in understanding how these forces interact has been made through the study of abstract models. Further progress may be made by identifying a model system in which the interaction between self-organization and selection can be investigated empirically. To this end, we investigate how the self-organizing thermoregulatory huddling behaviours displayed by many species of mammals might influence natural selection of the genetic components of metabolism. By applying a simple evolutionary algorithm to a well-established model of the interactions between environmental, morphological, physiological and behavioural components of thermoregulation, we arrive at a clear, but counterintuitive, prediction: rodents that are able to huddle together in cold environments should evolve a lower thermal conductance at a faster rate than animals reared in isolation. The model therefore explains how evolution can be accelerated as a consequence of *relaxed selection*, and it predicts how the effect may be exaggerated by an increase in the litter size, i.e. by an increase in the capacity to use huddling behaviours for thermoregulation. Confirmation of these predictions in future experiments with rodents would constitute strong evidence of a mechanism by which self-organization can guide natural selection.

## 1. Introduction

In a self-organizing system, a complex group behaviour emerges from local interactions between individuals behaving without plan or instruction (see [1]). Huddling behaviours displayed by mice [2], rats [3] and other social rodents [4], as well as penguins [5] and social insects [6], have been described formally as examples of self-organization. Agent-based computer modelling has demonstrated how group aggregation patterns can emerge, based on simple interactions between individuals. One such model [7] reveals how aggregation patterns observed in rodents exposed to different temperatures can emerge spontaneously when cold or warm (‘homeothermotaxic’) individuals simply turn towards warmer or colder littermates, respectively.

The aim of this study is to investigate the effects that self-organizing thermoregulatory huddling behaviours displayed by many mammals, birds and other social animals might have on the evolution of genetic components of thermoregulation. Thermoregulatory huddling is a self-organizing system with the advantage of being both simple to experimentally manipulate, and well-described by established theoretical and computational models.

Despite this, the potential for behavioural thermoregulation to affect evolution by reducing the metabolic costs of thermoregulation have been expressed only informally (e.g. [8]). When evolutionary algorithms have been applied to formal models of huddling, they have concentrated on fitting parameters to empirical data rather than formalizing evolutionary theory (e.g. [9]).

Here, a simple evolutionary algorithm is challenged to minimize metabolic cost by evolving two model genes, which specify physiological and morphological components of thermoregulation, respectively. We show that evolution occurs only when within-lifetime adaptability is introduced, i.e. when huddling is possible. Specifically, the model predicts that increasing within-lifetime adaptability by increasing the number of available huddlers should accelerate the evolution of physiological thermoregulation. The main result of this paper is the counterintuitive finding that cold-exposed animals which are allowed to huddle should evolve insulative fur and/or subcutaneous fat at a faster rate than animals reared in isolation.

We normally think of evolution by natural selection as a direct response to selection pressure. However, the model suggests that the evolution of thermal physiology may actually be improved when selection pressure on the growth of fat and fur is relaxed due to huddling.

## 2. Model

Thermoregulation is a complex emergent property of interactions between many factors affecting the metabolism of an organism. These can be broadly categorized as: (i) environmental factors including the climate and temperature around an organism, (ii) physiological factors regulating the capacity of the organism to generate heat, (iii) morphological factors determining the rate at which heat is lost from the body to the environment, and (iv) behavioural factors by which an organism may relocate or adapt its exposed surface area to regulate heat loss. Given the importance of thermoregulation for all biological processes [10], and the energetic costs of metabolism [11], we should expect interactions between environment, physiology, morphology and behaviour to play a central role in the evolution of species by natural selection [12].

In mammals, the amount of fat or fur imposes physiological and morphological limits on heat generation and heat loss. The relationships between these factors have been well characterized for endotherms, in particular via experiments with rodents such as rats and mice, from which we derive the majority of our modelling assumptions. Newton’s law of cooling [13–15] (see also [16]) can be used to derive an expression for metabolic rate, *M*

2.1 $$M=AC(T_{b}-T_{a}),$$

where *T*_b_ is the body temperature and *T*_a_ is the environment (or ambient) temperature; *C* is the whole body thermal conductance, which modulates the rate at which the body exchanges heat with the environment; and *A* is the proportion of the body surface area that is exposed to the ambient temperature. Equation (2.1) therefore formalizes the intuition that metabolic costs are greatest when a highly exposed body conducts heat rapidly to a cold environment.

The metabolic costs of prolonged cold exposure can be reduced by insulating the body to reduce the morphological factor *C*, or by moving or morphing the body to reduce *A*. Changes to both *C* and *A* can occur through several mechanisms (depending on the species and the environment) and on multiple timescales, as explained in the Discussion (see [17] for a comprehensive review). A simplifying assumption represented by the present model is that a change in *A* corresponds to an immediate behavioural change, whereas a change in *C* would occur predominantly on an inter-generational timescale. When the option is available, rodents are expected to respond to environmental change by adapting their behaviour rather than their physiology or morphology [18].

In cold environments, huddling allows each huddler to exploit the heat generation of others, to increase *T*_a_ in its local microclimate, and to reduce *A* [19–22]. Huddling allows an individual to reduce the proportion of its surface area that is exposed up to a limit that depends on the number *n* of aggregated animals. Derived from geometrical considerations, it has been found that *A* has a lower limit which varies with *n*, specifically,

2.2 $$n^{-1/4}\leq A\leq 1.$$

According to equation (2.2), the proportion of the surface area that is exposed has an upper bound of 1 (i.e. when the entire body is exposed), and a lower bound of *n*^−1/4^ (i.e. as the number of available huddlers *n* increases, the minimum exposed surface area that they can achieve, on average, by huddling together, decreases exponentially).

The degree to which increases in *n* can reduce the exposed surface area of the group varies depending on the geometry and morphology of the species. Estimates of the exponent −1/4 vary depending on the underlying geometrical assumptions about animal aggregation patterns [2]. In practice, an exponentially decaying function of *n*, such that metabolic savings asymptote for larger *n*, is supported by several investigations of aggregation in small mammals ([16,21,23–25]; see also [26]).

In a self-organizing model of rodent huddling [7], the thermoregulatory capacity of simulated huddles was shown to be greater than that of the individuals, and self-organization was found to yield adaptations of *A* in the average huddler consistent with a mathematical description of the huddle as a ‘super-organism’ able to thermoregulate by shifting its overall shape.

Accordingly, self-organizing behavioural interactions allow the litter to adapt the average area *A*(*n*) to extend the range of *T*_a_ over which *T*_b_ can be maintained at the preferred temperature, *T*_*pref*_, while *M* and *C* remain constant within a generation. The geometrical constraints defined by equation (2.2), combined with the ability of the huddle to adapt its surface area *A*(*n*) by self-organization according to the derivation of [7], yields the following relationship:

2.3 $$A(n)=\left\{ \begin{array}{ll} 1 & \text{if }1\leq A_{\mathrm{pref}}\\ A_{\mathrm{pref}} & \text{if }n^{-1/4}<A_{\mathrm{pref}}<1\\ n^{-1/4} & \text{if }A_{\mathrm{pref}}\leq n^{-1/4} \end{array}\right.,$$

where *A*_pref_=*M*/*C*(*T*_pref_−*T*_a_) is the exposed surface area required to maintain the body temperature at the preferred temperature.

The adaptable surface area defined by equation (2.3) can be substituted back into equation (2.1) to define the average body temperature for a litter of size *n*

2.4 $$T_{b}(n)=\frac{M}{CA(n)}+T_{a}$$

This allows a simple fitness function to be defined,

2.5 $$F=\left\{ \begin{array}{ll} \frac{M_{\max}-M}{M_{\max}} & \text{if }T_{b}(n)=T_{\mathrm{pref}}\\ 0 & \text{if }T_{b}(n)\neq T_{\mathrm{pref}} \end{array}\right.,$$

where *M*_max_ sets an upper bound on the metabolic rate. Litters able to maintain the average body temperature at the preferred temperature have a fitness which decreases with metabolic rate, and litters unable to maintain the average body temperature at the preferred temperature have a fitness of zero. In summary, equation (2.3) states how the size of the litter determines the extent to which the exposed surface area can be adapted by huddling, equation (2.4) specifies the resulting body temperature, and equation (2.5) incorporates the body temperature into the definition of a fitness function that promotes homeothermy and penalizes higher metabolic rates.

To investigate how the capacity for behavioural thermoregulation could affect selection of genes determining the limits of physiological and morphological thermoregulation, we can modify *M* and *C* using a simple evolutionary procedure. This procedure is used to evolve a population comprising *N* litters, where each litter is represented as a pair of metabolic rate and thermal conductance values. For convenience, the population is maintained as *N* pairs of *m* and *c* values ranging 0–1, scaled to obtain *M*=*M*_max_*m* and *C*=*C*_max_*c* when equation (2.5) is used to determine fitness (this allows a single parameter *σ* to specify comparable effects of mutation for both genes).

To make each child litter, two different parent litters (mum and dad) are chosen from the population at random with a probability proportional to their relative fitnesses. Each generation is populated by repeating the following process of recombination *N* times (hence each parent may seed multiple children). First, the metabolic rate of the child litter is chosen to fall randomly between bounds set by the two parent values

2.6 $$m=r_{1}m_{\mathrm{mum}}+(1-r_{1})m_{\mathrm{dad}},$$

where *r*_1_ is a random number from the uniform distribution *r*_1_∈[0,1]. This value of *m* is then modified for mutated genes by setting *m*=*m*+*r*_2_, where *r*_2_ is a random number from a uniform distribution *r*_2_∈[−*σ*,*σ*]. The value of *c* for each child is determined in exactly the same way, from the same parents and with *r*_1_ and *r*_2_ generated anew, and the two genes are mutated (independently) with a fixed probability, set to *p*=0.1 here.

The effect of mutation (for genes that are selected to mutate) was set to *σ*=0.1, the ambient temperature was set to *T*_a_=20°C and the preferred temperature was set to *T*_pref_=37°C, the maximum metabolic rate was set to *M*_max_=37 kcal d^−1^, the maximum thermal conductance was set to *C*_max_=2 kcal (d°C)^−1^, and the population size was set to *N*=500 litters. Note that the behaviour of the model is robust to changes in the value of *T*_a_, for ambient temperatures below *T*_pref_, and it is robust to variation of the population size. The value chosen for *M*_max_ represents the intuitive assumption that a mutation in metabolic rate which causes the body temperature to exceed *T*_pref_ (when fully exposed to ambient temperatures above 0°*C*) cannot be viable.

To help explain the behaviour of the model, it is useful to define the boundary conditions that separate litters with zero fitness from litters with non-zero fitness, as expressed in equation (2.5). We can do this by substituting *T*_b_=*T*_pref_ into equation (2.1)

2.7 $$M=(T_{\mathrm{pref}}-T_{a})CA(n).$$

In essence, this states that non-zero fitness is achievable when the genetically specified values of *M* and *C* allow *A*(*n*) to be varied so as to keep the body temperature at *T*_pref_.

Equation (2.7) effectively defines two boundaries. At one boundary, *A*_pref_≥1, so *A*(*n*)=1, and therefore *M*=(*T*_pref_−*T*_a_)*C*. At this boundary, the exposed surface area can increase no further because all pups in the litter are isolated, and any increase in ambient temperature will cause their body temperatures to exceed the preferred temperature.

At the other boundary, *A*_pref_≤*n*^−1/4^, so *A*(*n*)=*n*^−1/4^, and therefore *M*=(*T*_pref_−*T*_a_)*Cn*^−1/4^. At this boundary, the exposed surface area can decrease no further because the litter is maximally huddled, and any further reduction in ambient temperature will cause the average body temperature to drop below the preferred temperature.

For the ‘no-huddling’ control condition, combinations of *M* and *C* that yield non-zero fitness are confined to the solution of equation (2.7), when *n*=1. To reveal the evolutionary dynamics, it is therefore convenient to initialize populations with values of *M* and *C* such that some in the initial population have a chance of non-zero fitness. Populations of litters were thus initialized with uniformly distributed random values ranging *m*∈[0.8−*σ*/2,0.8+*σ*/2] and *c*∈[0.8−*σ*/2,0.8+*σ*/2].

An implementation of the model (written in Python 2.7) is available as electronic supplementary material, S1 and S2.

## 3. Results

An evolutionary algorithm was used to test how natural selection for the morphological and physiological components of thermoregulation might be affected by a capacity for self-organizing huddling behaviour to support within-lifetime adaptation to a cold environment. Populations of litters of various sizes were evolved under explicit pressure to, (i) maintain the average body temperature of the litter at *T*_pref_ and, (ii) minimize the metabolic rate, and thus the metabolic cost of thermoregulation. The metabolic rate *M* and thermal conductance *C* were subjected to natural selection. Crucially, lower metabolic rates were explicitly associated with lower cost, but lower thermal conductances were not. Within each simulation, the litter size *n* was kept constant across litters, and the effects of increasing *n* were compared between simulations. Many randomly seeded populations through a full range of litter sizes were evolved for several thousand generations each, and the dynamics summarized next were observed to be highly robust.

**Figure 1.**
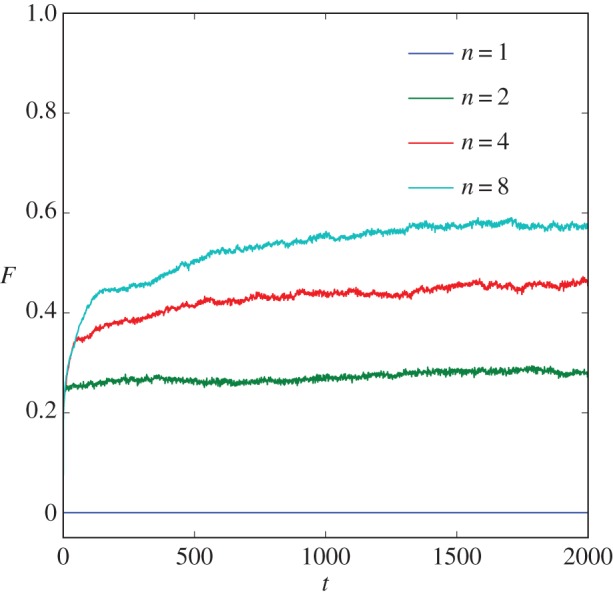
Behavioural thermoregulation accelerates the evolution of physiological thermoregulation. Populations of litters, each specified genetically as a combination of a metabolic rate and a thermal conductance, were evolved to minimize metabolic costs while maintaining a stable body temperature. Each line shows how thermoregulation evolves in populations comprising litters of a given size *n*. The average fitness *F* of the population is plotted against time *t* (in generations). In the no-huddling control condition (*n*=1), fitness does not increase. However, for litters that can adapt to the environment by huddling (*n*>1), fitness increases over time. The model predicts that as the capacity for adaptation by self-organizing huddling increases (i.e. as litter size *n* increases) so too will the rate of evolution of genes specifying the physiological and morphological components of thermoregulation. See figure 2 for a mechanistic account of these effects.

Figure 1 shows how the average fitness in the population evolves over time for four example populations comprising litters of size *n*=1, *n*=2, *n*=4 and *n*=8, respectively. In the no-huddling control condition (*n*=1), fitness did not increase over time. However, for litters able to adapt by huddling (*n*>1), the population fitness increased steadily. Populations comprising larger litters evolved more quickly and reached higher asymptotic fitness.

**Figure 2.**
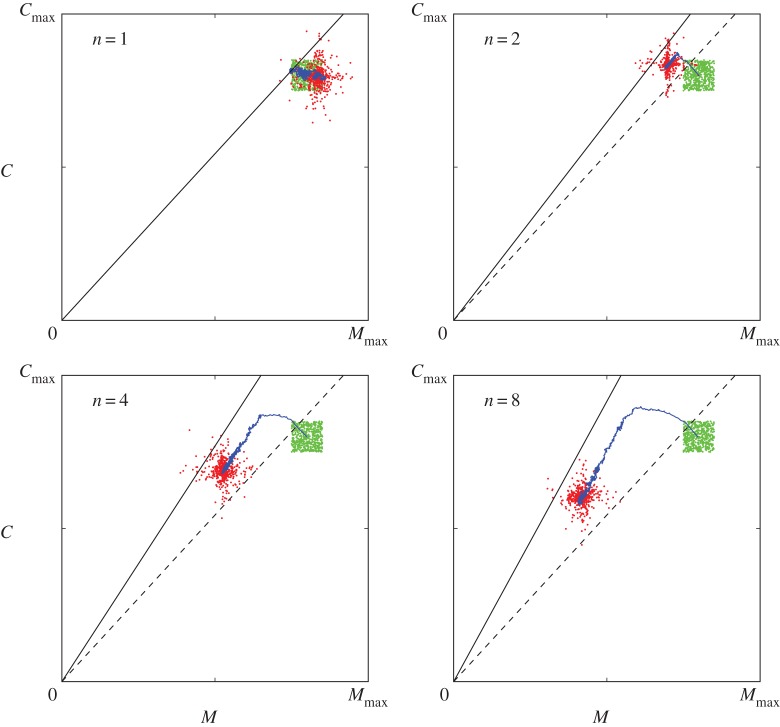
Evolution of thermoregulation in the fitness landscape. Results from the simulations reported in figure 1 are shown. Each panel depicts the evolution of thermoregulation in a population comprising litters of a given size, *n*. Solid and dashed straight lines indicate the lower and upper boundaries of a ‘zone of increased fitness’, within which the litter is able to maintain the average body temperature at a preferred temperature by within-lifetime (behavioural) thermoregulation, i.e. by huddling. The initial population is shown as a square-shaped cluster of green dots, the trajectory of the population average is shown as a continuous blue line, and the final population after 2000 generations is shown as a cluster of red dots. In the control condition, where *n*=1 and hence huddling is impossible, the zone of increased fitness is almost impossible to find by chance, hence the initial and final populations are indistinguishable except for the drift of a random walk and the effects of mutation. However, as *n* increases, the capacity for huddling makes the zone of increased fitness easy to find. When the population enters this zone, explicit selection pressure to minimize *M* pushes the population to the left of the landscape, and as the upper boundary is approached, indirect selection based on the failure of litters straying beyond it push the population down the landscape. Interestingly, the evolutionary dynamics also minimize the thermal conductance *C* despite no explicit metabolic cost or selection pressure being associated with this component of thermoregulation in the fitness function.

To understand how self-organizing thermoregulatory huddling can accelerate evolution, it is useful to study the trajectory of each population as it evolves through the two-dimensional (*M*,*C*) fitness landscape. Each panel in figure 2 shows the trajectory of a single population, with the initial and final generations connected by a blue line depicting the change in the population average over time. Note that in each condition, including the *n*=1 control, the distribution of the final population in the fitness landscape tends from an initial square shape to a cross shape, simply because the probability *p*^2^ of both genes mutating in a given child litter is less than the probability 2(*p*−*p*^2^) that only one gene mutates. Otherwise, any differences between the initial and final generations in the control condition merely reflect the random walk of the population through the fitness landscape.

Two lines in each panel in figure 2 represent solutions to equation (2.7) for *A*=1 (dashed line), below which litters overheat, and for the geometrical limit of huddling, *A*=*n*^−1/4^ (solid line), above which litters are too cold to maintain the preferred body temperature. Between these boundaries, litters are able to maintain *T*_b_=*T*_pref_ by adapting *A* (i.e. by huddling), hence the region defined by 1≤*C*(*T*_pref_−*T*_a_)/*M*≤*n*^1/4^ constitutes a ‘zone of increased fitness’ [27].

Once the population enters this zone of increased fitness, it is subject to an explicit pressure to minimize *M*, and (as expected) the metabolic rate decreases. Note that the initial reduction in *M* occurs at the same rate for all *n*>1, although it continues for longer in the wider zones of larger litters, thus accounting for the similar rates of initial fitness increase shown for each litter size in figure 1. In each case, *M* drifts freely with respect to *C*, with which no metabolic cost or other selection pressure had been explicitly associated (see equation (2.5)). However, when the population encounters the lower bound on the metabolic rate for a given thermal conductance, the conductance also starts to fall, and the reduction of *M* and *C* becomes correlated. In figure 2, the combination of *M* and *C* can be seen to evolve with a trajectory that runs parallel to the upper boundary of the zone of increased fitness.

Directed evolution of the thermal conductance, in the absence of an explicit selection pressure on *C*, is an interesting and surprising result, but figure 2 reveals the underlying mechanism to be straightforward. At the upper boundary of the zone of increased fitness, litters that fail to maintain the preferred body temperature due to a low metabolic rate do not survive, preventing further reduction of *M*. Similarly, litters that (by chance mutation) have a high thermal conductance may stray outside the zone of increased fitness, biasing the population towards lower thermal conductances. At lower thermal conductances, the potential for the explicit selection for low metabolic rates to reach still lower rates is greater, hence the reduction of *M* continues to be complemented by a reduction in *C* away from the upper boundary. Explicit selection for litters with lower metabolic rates *M*, due to the appearance of *M* in the fitness function (equation (2.5)), continually pushes the population towards the upper boundary, above which litters are too cold. At this boundary, selection based on the failure of high thermal conductances is implicit (because *C* does not appear as a term in the fitness function); this effectively pushes the population away from the boundary to regions in the fitness landscape where the *potential* for further reduction in the metabolic rate is greater. The net effect is that the population maintains a distance from the upper boundary, and this distance is determined by the rate and extent of mutation.

This continues as the zone of increased fitness narrows, until mutation pushes some litters in the population below the lower boundary where they overheat, at which point evolution effectively stops. Zones of increased fitness defined by larger *n* are wider, and therefore larger litters evolve lower values of *M* and *C*. This increased width of the zone of increased fitness associated with larger litters (*n*) accounts for the increased asymptotic fitness of larger litters (figure 1).

## 4. Discussion

A simple evolutionary algorithm was challenged to discover a combination of genes to specify the physiological and morphological components of thermoregulation, and to optimize the former to reduce metabolic cost. The challenge was such that, in the case where individuals cannot huddle, and hence adaptation of the exposed surface area is not possible, solutions where homeothermy can be achieved were constrained to a very narrow region of the fitness landscape. A valid solution is combinatorially difficult to find by random search alone, like finding a needle in a haystack (see [27]). Moreover, should a solution be found, reducing metabolic costs by random local search is difficult because any mutation in metabolic rate is catastrophic unless paired with a precise compensatory mutation in thermal conductance, so optimization becomes like taking a random walk along a tightrope. Fitness landscapes defined with physiological and morphological tolerances in mammals may of course be more forgiving than in the model, but consideration of the most treacherous landscape used here is useful for exposing the full potential for within-lifetime adaptation to guide evolution.

According to the model, allowing the exposed surface area to adapt during the lifetime increases the range of thermal conductances over which a preferred body temperature can be maintained for each genetically specified metabolic rate. Crucially, as the population evolves, no information is directly communicated from phenotype to genotype, yet over generations the thermoregulatory effects afforded by within-lifetime adaptation of the exposed surface area becomes consolidated as a genetic adaptation in the thermal conductance. Evolution proceeds until the advantages of huddling are offset by the extent of mutation, hence the final population retains a degree of within-lifetime adaptability.

McNab [12] considers that small endothermic mammals (with low thermal conductance and high mass-specific metabolic rates) were unlikely to have evolved directly from small ectothermic reptiles (with high conductances and low mass-specific metabolic rates) because intermediate stages would not have been viable; ‘… a small endotherm with reptilian conductance would squander heat in a hopeless attempt to maintain a constant body temperature’ [12]. Instead he proposed that reptiles ancestral to mammals first increased in body mass, gaining a degree of ‘inertial homeothermy’, i.e. a resistance to changes in temperature due to a reduced surface-area to volume ratio, before developing a fur coat that further improved the constancy of body temperature. Homeothermy afforded nocturnal hunting and/or foraging (see also [28]), and endothermic mammals subsequently emerged from an incremental (linear) reduction in thermal conductance and basal metabolic rate as body size reduced (and mass-specific metabolic rate increased). This account is corroborated by a transition from the lizard to mammalian lineages whereby metabolic rate and thermal conductance remain constant as intermediate species drifted with respect to body mass (compare figure 2 in this paper with fig. 2 in [12]). Interestingly, a model representing this proposal would be similar in form to that presented here, except that the adaptability parameter (i.e. the capacity for huddling, *n*) should instead be expressed in terms of the postulated inertia of pre-endothermic homeothermy. The common underlying mechanism is a relaxation of selection pressure on one parameter (i.e. thermal conductance) allowing another (i.e. metabolic rate) to drift freely, followed by an interaction with a boundary in the combined fitness landscape that correlates the subsequent evolution of both.

An alternative theory of the evolution of endothermy from ectothermy emphasizes the selective advantage of sustaining a high metabolic rate for aerobic exercise over thermogenesis, the latter providing only secondary benefits for thermoregulation that were exploited subsequently. Accordingly, direct selection for an increase in the maximal metabolic rate would reveal itself as an indirect selection for an increase in the resting metabolic rate [29]. This ‘aerobic theory’ is supported by recent population genetic analyses confirming the central prediction that maximal and resting metabolic rates should be associated by a high genetic correlation [30].

According to [29], selection for thermoregulation over aerobic capacity would have acted only to reduce thermal conductance by optimizing the growth of insulative fat or fur, keeping the costs of sustaining a high resting metabolism to a minimum [29]. It is therefore interesting that significantly high heritability and high additive genetic variance of thermal conductance have been reported in cold-acclimated mice, suggesting that thermal conductance is a potential target for natural selection in this species [31]. Furthermore, in a follow-up study with the same species, basal metabolic rate and maximum metabolic rate were found not to be significantly heritable [31,32]. Instead, the authors reported a high (negative) genetic correlation between birth mass and non-shivering thermogenesis, i.e. brown adipose tissue (BAT) thermogenesis, more consistent with an inertial homeothermy than an aerobic account for these highly social (wild-caught) rodents. In their words ‘… this is an interesting outcome since it relates adult capacity for aerobic energy expenditure to a very different attribute, related to the quality of pups in a litter. … In other words, non-shivering thermogenesis could respond to indirect selection on birth mass’ [32]. Might huddling provide the basis for this indirect relationship?

BAT-thermogenesis is thought to be critical for effective huddling in rodents, as evidenced by experiments showing that rats move to cooler locations when BAT is pharmaceutically increased [33], and that huddling in rats ceases when BAT is pharmaceutically blocked [34]. Interestingly, Syrian golden hamsters, which are born without functional BAT [35], and do not huddle until BAT becomes functional at around postnatal day 14, have been shown to huddle when fostered into litters of weight-matched rats with functional BAT [36]. These data are consistent with the central role of BAT thermogenesis in the self-organization of rodent thermoregulatory huddling behaviours, according to the model of [7].

A recent study found no relationship between BAT-thermogenesis and birth weight in neonatal rabbits [37]. However, pups born heavier are known to occupy the warmer central positions in the huddle, whereas lighter pups occupy the cooler peripheral positions [38]. Pups who spent more time at the periphery of the huddle responded to an acute cold challenge at postnatal day 3 with a greater reduction in BAT metabolism compared with pups that occupied the centre of the huddle ([37]; see also [39]). Similar huddling patterns have been reported for lighter pups cross-fostered to be heavy relative to their surrogate littermates, with relatively heavy littermates occupying the warm huddle centre. Hence, any relationship between birth weight and adult thermogenesis may indeed be an indirect one, mediated by the thermotaxic struggle for position in the huddle. In circumstantial support, lighter (adult) rats move to higher ambient temperatures in a thermocline than heavier rats, where they are found to metabolize at higher rates [40].

Furthermore, the effects of birth weight on many aspects of later development are similar to the effects of litter size; for example pre-weaning weight gain and the development of motor coordination are both improved in heavier pups [41,42], but they are also improved in pups raised in litters compared with those reared in isolation [43]. We might speculate that since first exploiting the thermoregulatory benefits of huddling, evolution may have later exploited a variety of possible benefits of social thermoregulation for later development [44–48].

Huddling has been considered as an epigenetic factor in several other discussions. For example, Haig [8] explains that the genes encoding the potential for BAT-thermogenesis (Pref1/Dlk1 and Necdin) interact with a BAT-activating gene (GNAS) either to promote thermogenesis when the maternal allele is expressed or to inhibit thermogenesis when the paternal allele is expressed, and therefore males and females have a differential genetic investment in the success of the huddle. In support, male rat pups, who generate less heat through BAT, have been described as heat sinks that drain the extra heat generated by female littermates [49]. These authors also suggest that the ratio of males to females in a litter can affect BAT-thermogenesis directly via sex hormones, hence the adaptive capacity for huddling may be affected by the sex ratio within the huddle. Another potential epigenetic effect is suggested by the experiment of Yamauchi *et al.* [50], who bred from mice housed (in pairs) under two conditions; the first from parents housed at a fixed ambient temperature from 8 weeks that mated between 9 and 11 weeks, and the second from parents who were temperature controlled from 8 weeks and mated between 16 and 18 weeks. Mice maintained at ambient temperatures above 27°C bore litters averaging around 9 pups, whereas mice maintained at lower temperatures had litters averaging upwards of 11. Remarkably, the animals housed below 27°C for the longer period had litters averaging around 15 pups. Thus, prolonged cold-exposure in one generation can increase the litter size, and hence increase the capacity to adapt to the cold by huddling, in the next generation. It would be interesting in an extension of the model to establish the potential implications of these epigenetic effects for the evolution of endothermy.

We might have naively expected that cold-exposed animals with the opportunity to keep warm by huddling should evolve a reduced thermal conductance at a slower rate than animals reared in isolation. But the present model clearly makes the opposite prediction; by reducing selection pressure on the thermal conductance, huddling allows cold-exposed populations to reduce thermal conductance at a faster rate compared with non-huddlers. The mechanism behind this effect therefore corresponds to what Deacon refers to as ‘relaxed selection’ [51], whereby outsourcing selection pressure to the environment relaxes the selection pressure on the two genetic components of thermoregulation, freeing one to vary independently of the other.

This study has been concerned with establishing the potential for self-organizing behaviour within the lifetime to alter the course of evolution. The experimental test of the model would be to breed successive generations of rodents reared in cold environments; animals bred and reared in larger groups should evolve insulative fur and/or subcutaneous fat faster than those reared in smaller groups.

Self-organizing thermoregulatory huddling is probably one of several within-lifetime factors that can help accelerate the evolution of endothermy by natural selection. Establishing the relative contribution of each factor in controlled experiments may help reveal in more general terms the extent to which self-organization guides evolution.

## Supplementary Material

Code required to run the simulations and reproduce the Figures has been uploaded as part of the supplementary material. S1. Implementation of the model in c++. The model can be compiled from the command line using the command [g++ model.cpp -o model] and it can then be run using the command [./model 1 8 500 2000 20.0 37.0 2.0 0.8 0.1 0.1] to generate data for a population comprising n = 8 litters.

## Supplementary Material

Code required to run the simulations and reproduce the Figures has been uploaded as part of the supplementary material.S2. Analysis of the data in Python. After compiling S1, this script can be launched from the same directory to re-generate Figure 1 and Figure 2 from the main text (uses NumPy and matplotlib libraries).

## Data accessibility

All data associated with this manuscript are included in the electronic supplementary material (see Data, code and materials).
